# Automated measurement of coagulation and fibrinolytic activation markers: Outcomes in coronavirus disease 2019 (COVID‐19) patients

**DOI:** 10.1111/ijlh.13855

**Published:** 2022-04-22

**Authors:** Chris Gardiner, Ian J. Mackie, Peter MacCallum, Sean Platton

**Affiliations:** ^1^ Specialist Laboratory Medicine St James University Hospital, Leeds Teaching Hospitals NHS Trust Leeds UK; ^2^ Haemostasis Research Unit University College London London UK; ^3^ Wolfson Institute of Population Health Queen Mary University of London London UK; ^4^ Department of Haematology Barts Health NHS Trust London UK; ^5^ The Royal London Haemophilia Centre Barts Health NHS Trust London UK; ^6^ NHS East and South East London Pathology Partnership Barts Health NHS Trust London UK

**Keywords:** chemiluminescent assays, COVID‐19, fibrinolysis, thrombomodulin, vascular endothelium

## Abstract

**Background:**

Severe coronavirus disease 2019 (COVID‐19) is characterized by marked hypoxaemia and lung oedema, often accompanied by disordered blood coagulation and fibrinolytic systems, endothelial damage and intravascular fibrin deposition.

**Patients/Methods:**

We present a retrospective observational study of 104 patients admitted to hospital with COVID‐19. Plasma samples were collected within 72 h of admission. In addition to routine coagulation and haematology testing, soluble thrombomodulin (sTM), thrombin‐antithrombin (TAT), tissue plasminogen activator‐plasminogen activator inhibitor 1 complex (tPAI‐C) and plasmin‐α2 antiplasmin complex (PIC) were performed by automated chemiluminescent enzyme immunoassays.

**Results:**

Significantly higher levels of D‐dimer, TAT, sTM and tPAI‐C were observed in non‐survivors compared to survivors. To confirm which parameters were independent risk factors for mortality, multiple logistic regression was performed on D‐dimer, TAT. sTM, tPAI‐C and PIC data. Only increasing sTM was significantly associated with mortality, with an odds ratio of 1.065 for each 1.0 TU/mL increment (95% CI 1.025–1.115).

**Conclusions:**

Of the haemostatic variables measured, sTM, which can be rapidly assayed, is the best independent predictor of mortality in patients hospitalized with COVID‐19, and this suggests that endothelial dysfunction plays an important role in disease progression.

## 1 INTRODUCTION

Coronavirus disease 2019 (COVID‐19) is the severe acute respiratory syndrome caused by coronavirus‐2 (SARS‐CoV‐2). Severe disease is characterized by marked hypoxaemia and lung oedema, often accompanied by disordered blood coagulation and fibrinolytic systems, endothelial damage and intravascular fibrin deposition.^1^ Coagulation abnormalities are associated with poor prognosis.^2^ Pulmonary immunothrombosis^1^ and venous thromboembolic disease^3^ are common in hospitalized patients with COVID‐19. Despite this, overt disseminated intravascular coagulation, as defined by International Society on Thrombosis and Haemostasis criteria,^4^ is uncommon in patients with COVID‐19,^5^, ^6^ with little evidence of consumptive coagulopathy in most patients.

Increased D‐dimer, with longer prothrombin time (PT) and activated partial thromboplastin time (APTT) on admission are associated with increased mortality.^2^ Several authors have reported that increased levels of tissue plasminogen activator inhibitor‐1 (PAI‐1),^7^, ^8^, ^9^ tissue plasminogen activator (tPA)^7^ and soluble thrombomodulin (sTM)^7^, ^9^ are associated with increased mortality. However, most of the methods used were enzyme‐linked immunoassays (ELISAs), which are not suitable for rapid testing. We hypothesized that rapid automated assays of coagulation and fibrinolytic activation markers may provide additional information on the coagulopathy associated with severe COVID‐19.

We present a retrospective observational study in which rapid automated coagulation and fibrinolytic activation markers were analysed by chemiluminescent enzyme immunoassays (CLIEA), on plasma samples in addition to routine haematology testing, collected within 72 h of admission from patients admitted to hospital with COVID‐19.

## 2 MATERIALS AND METHODS

A total of 393 citrated plasma samples received by the coagulation laboratory at the Royal London Hospital (RLH) for routine analysis, were collected between 4 April 2020 and 31 December 2020 from patients with clinical details containing “COVID” or “corona.” Due to the pressures of the pandemic, it was only possible to save a limited number of samples and samples were not collected from consecutive patients (over 15 000 patients with COVID have been admitted to Barts Health Hospitals during the pandemic).

All samples were centrifuged within 1 h of collection, and plasma was double‐spun, separated and frozen below −70°C within 4 h. The samples were anonymized after recording the sex, age, ventilation status, SARS‐Cov‐2 PCR results, routine haematology results, date of admission and date of discharge or death. It was not possible to follow up patients after discharge. The saved plasma used does not fall under the auspices of the Human Tissue Act,^10^ and the use of anonymized material did not require ethics approval according to Medical Research Council guidance^11^.

For the current study, only samples collected within the first 72 h after admission were selected and, if several samples from the same patient were available, only first sample was used. In total, 104 samples met these criteria, with 37 collected in the first 24 h, 60 from 24 to 48 h, and 7 from 48 to 72 h after admission. The date of onset of symptoms was not recorded for the purposes of this study.

Patients were divided into three groups based on their ventilation status at the time of sample collection: not ventilated, receiving continuous positive airway pressure (CPAP) or mechanically ventilated (MV). Decisions to use CPAP and mechanical ventilation were individualized, and escalation was based on deteriorating response to supplemental oxygen. Although many of the patients were likely to have received low molecular weight heparin (LMWH), as per local protocol, the investigators were unaware of which patients were receiving prophylactic, intermediate or therapeutic doses of LMWH. However, anti‐Xa heparin levels were measured on the study samples.

Full blood counts were performed on the Sysmex XN‐9000 haematology analyser (Sysmex Corp. Kobe, Japan). Routine clinical coagulation tests were performed on Sysmex CS5100 analysers (Sysmex Corp) using standard protocols. The following reagents were used: Prothrombin time (PT) Dade® Innovin®, activated partial thromboplastin time (APTT) Siemens Actin® FS, Clauss fibrinogen Dade Thrombin Reagent, INNOVANCE® D‐dimer, INNOVANCE Antithrombin (all Siemens, Marburg, Germany) and Biophen LRT Heparin (Hyphen Biomed, France). Thrombin‐antithrombin (TAT), soluble thrombomodulin (TM), tissue plasminogen activator‐plasminogen activator inhibitor 1 complex (tPAI‐C) and plasmin‐α2 antiplasmin complex (PIC) were performed using CLEIA (Sysmex) on the Sysmex CN‐6500 analyser as previously described.^12^

GraphPad Prism 9.1 (GraphPad Software, California, USA) was used for all statistical analysis. Nonparametric methods were used throughout (Mann–Whitney *U* test for numerical variable or Fisher's exact test for the analysis of contingency tables).Receiver operator curve (ROC) was used to assess the discriminative capacity of coagulation and CLEIA test results. Odds ratios were calculated using the Baptista Pike method. Multivariate analysis was performed using multiple logistic analysis and principal component analysis (PCA).

## 3 RESULTS

The median age of the 104 patients from whom the samples were analysed was 58 years and 48% were female (Table 1). At the time of sample collection 20 were receiving MV, 34 were on CPAP and 50 were unventilated. Ventilation status had no significant influence of the time between admission and sample collection (median 1 day for all groups).

**TABLE 1:** Patient characteristics at admission (<72 h) by ventilation status at the time of sampling. Data are expressed as median and interquartile range. Statistical differences between groups are expressed as *P* values (Mann Whitney *U* test or Fisher's exact test). Reference intervals for CLEIA were: sTM 6.9–14.5 TU/mL, TAT 0.40–3.25 ng/mL, tPAI‐C 2.23–18.72 ng/mL and PIC 0.293–1.319 μg/mL

Variable	Ventilated (*n* = 20)	CPAP (*n* = 34)	Not ventilated (*n* = 50)	*P* value ventilated v CPAP	*P* value ventilated v not ventilated	*P* value CPAP v not ventilated
Age	59 (44–73)	66 (46–72)	49 (42–65)	NS	NS	NS
Female	11 (55%)	17 (50%)	22 (44%)	NS	NS	NS
Days in hospital	13 (9–20)	6 (4–13)	12 (1–19)	NS	NS	NS
PT (s)	11.4 (10.9)	11.3 (10.8–12.6)	10.9 (10.5–11.5)	NS	.03	.005
APTT (s)	27.4 (25.2–32.9)	28.6 (25.0–32.1)	25.4 (24.1–29.5)	NS	NS	NS
Fibrinogen (g/L)	5.33 (3.74–6.32)	6.22 (4.77–7.66)	5.40 (4.45–6.68)	NS	NS	NS
D‐dimer (mg/L)	6.55 (2.08–42.27)	1.56 (0.80–5.680	0.71 (0.42–0.94)	.008	<.0001	.0003
FDP (mg/mL)	13.9 (6.7–81.5)	4.9 (2.9–17.8)	2.7 (2.5–4.1)	.02	<.0001	.002
Antithrombin (IU/mL)	79.0 (72.8–100.7)	95.4 (84.6–107.4)	96.6 (83.0–104.9)	NS	NS	NS
WBC	9.2 (7.3–14.8)	8.4 (5.4–11.5)	6.8 (4.9–8.8)	NS	.003	.008
Neutrophils x10^9^/L	8.28 (5.67–13.17)	6.91 (4.31–10.74)	5.49 (3.73–7.09)	NS	.01	.03
Lymphocytes x10^9^/L	0.77 (0.50–1.14)	0.75 (0.58–0.98)	0.91 (0.64–1.19)	NS	NS	NS
Platelets x10^9^/L	218 (143–282)	239 (167–304)	236 (190–328)	NS	NS	NS
TAT (ng/mL)	18.1 (13.2–24.9)	13.2 (9.0–22.1)	9.4 (7.9–16.6)	<.006	<.0001	<.0001
sTM (TU/mL)	25.7 (8.6–79.4)	9.1 (5.9–16.5)	4.5 (2.9–7.1)	.05	.0005	NS
tPAI‐C (ng/mL)	24.1 (13.5–40.7)	19.4 (15.5–34.1)	16.8 (12.3–20.5)	NS	NS	NS
PIC (μg/mL)	2.475 (1.320–15.766)	2.220 (1.716–3.383)	1.683 (1.17–2.449)	NS	.04	.04

D‐dimer, WBC and absolute neutrophil counts were highest in mechanically ventilated patients, and lowest in non‐ventilated patients (Table 1). Longer PTs were observed in CPAP and ventilated than non‐ventilated patients. This did not appear to be associated with abnormal liver function tests as otherwise reported by the laboratory. 29/104 samples (28%) had detectable anti‐Xa levels (median 0.25 IU/dL, range 0.11–1.61 IU/mL). Ventilation status had no significant effect on APTT, fibrinogen, antithrombin, lymphocyte count, or platelet count.

TAT levels were significantly higher in ventilated patients than patients receiving CPAP or non‐ventilated patients (Table 1) and were also significantly higher in patients receiving CPAP than non‐ventilated patients. Median sTM levels were higher in ventilated than the CPAP (2.5‐fold higher) and non‐ventilated patients (4‐fold higher), although the difference only achieved statistical significance between MV and non‐ventilated patients.

Overall 26.9% died including 40.0% of those on MV, 41.2% of those on CPAP and 12.0% of those not requiring advanced breathing support. Non‐survivors were significantly older than surviving patients (Table 2), but no significant differences in sex were observed. Longer PTs and APTTs were observed in non‐survivors but no significant differences in antithrombin or fibrinogen were seen. Non‐survivors had significantly higher WBC and neutrophil counts, but lower lymphocyte counts, than survivors. There was no significant difference in platelet count between survivors and non‐survivors.

**TABLE 2:** Patient characteristics by outcome. Data are expressed as median and interquartile range. Statistical differences between survivor and non‐survivor are expressed as P values (Mann Whitney *U* test or Fisher's exact test)

Variable	Survivors (*n* = 76)	Non‐survivors (*n* = 28)	Survivor v non‐survivor	AUC
Age	55 (42–69)	70 (48–79)	0.02	
Female	33 (43%)	17 (61%)	NS	
Ventilated	12 (16%)	8 (29%)	NS	
CPAP	20 (26%)	14 (50%)	NS	
Not ventilated	44 (58%)	6 (21%)	<0.0001	
Days in hospital	9 (5–15)	10 (6–116)	NS	
PT (s)	10.9 (10.5–11.7)	11.6 (10.9–12.8)	0.01	0.662
APTT (s)	25.9 (23.4–29.2)	32.5 (27.1–39.4)	<0.0001	0.771
Fibrinogen (g/L)	5.77 (4.36–6.82)	5.79 (3.9–6.85)	NS	0.509
D‐dimer (mg/L)	0.82 (0.49–2.00)	3.06 (1.35–10.32)	<0.0001	0.767
Antithrombin (IU/mL)	94.8 (81.5–104.9)	96.6 (69.85–107.2)	NS	0.515
WBC	7.03 (4.85–9.25)	11.00 (7.59–18.62)	0.004	0.682
Neutrophils ×10^9^/L	5.67 (3.67–7.36)	8.61 (6.11–13.20)	0.003	0.687
Lymphocytes ×10^9^/L	0.90 (0.65–1.16)	0.65 (0.50–0.85)	0.02	0.651
Platelets ×10^9^/L	239 (187–307)	224 (152–332)	NS	0.599
TAT (ng/mL)	5.8 (3.2–13.4)	10.1 (7.4–22.1)	0.006	0.677
sTM (TU/mL)	10.0 (7.8–14.1)	24.9 (17.9–42.5)	<0.0001	0.814
tPAI‐C (ng/mL)	16.5 (12.2–25.0)	26.9 (18.0–37.7)	0.02	0.658
PIC (μg/mL)	1.838 (1.201–2.850)	2.278 (1.542–6.446)	NS	0.620

Significantly higher levels of D‐dimer, TAT, sTM and tPAI‐C were observed in non‐survivors. ROC analysis demonstrated that increased sTM levels had the highest discriminatory capacity of the test parameters studied. Patients with sTM above the reference interval (*n* = 38) were more likely to die than patients with normal levels (*n* = 61) with an odds ratio of 11.32 (95% CI 4.01–33.2, sensitivity 88.9% and specificity of 59.2%) for mortality. ROC analysis showed that a D‐dimer cut off value of >0.8 mg/L had the best discriminatory function (sensitivity 92.6%, specificity 52.7%), which had an odds ratio of 7.52 (95% CI 2.19–24.81). Survivors with sTM above the reference interval on admission spent twice as long in hospital compared to those with normal levels (median 7 days vs. 15 days), although this was not statistically significant.

TAT and PIC were positively correlated with D‐dimer levels (*r* = .64 and *r* = .62 respectively), whereas neither sTM nor tPAI‐C demonstrated a significant relationship with D‐dimer levels.

**FIGURE 1:**
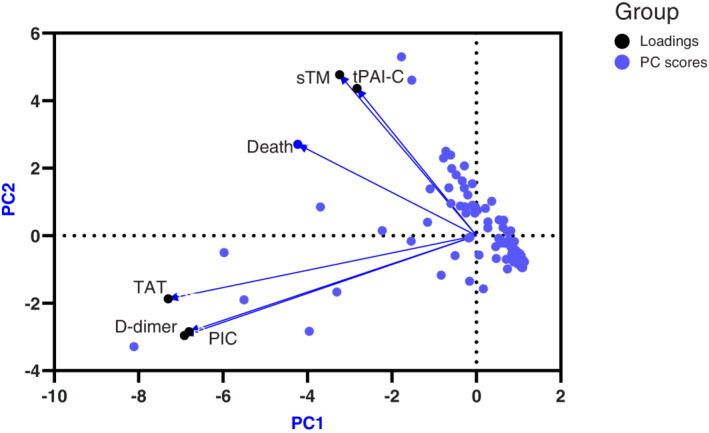
Principal component analysis of sTM, tPAI‐C, TAT, D‐dimer and PIC. Principal component scores are represented in blue and loadings in black

We analysed our data using PCA, a statistical pattern detection tool that reduces the dimensionality of a complex data set by means of a covariance matrix. This is used to create principal components, which are constructed as linear combinations of the initial variables in such a way that PCA1 explains the maximal amount of variance, while PCA2 accounts for the next highest variance. Thus PCA identifies clusters of variables that move together as groups. When applied to our data, PCA demonstrated that sTM and tPAI‐C were clustered, and were distinct from D‐dimer, TAT and PIC which were dependent on each other (Figure 1). To confirm which parameters were independent risk factors for mortality, multiple logistic regression was performed on D‐dimer, TAT. sTM, tPAI‐C and PIC data. Only increasing sTM was significantly associated with mortality, with an odds ratio of 1.065 for each 1.0 TU/mL increment (95% CI 1.025–1.115). Of the other haemostatic activation markers, only PIC came close to achieving statistical significance with an odds ratio of 1.170 (0.991–1.552) for each increment of 1.0 μg/mL.

## 4 DISCUSSION

We have presented the results of blood coagulation, haematology and CLEIA coagulation and fibrinolytic activation markers in samples collected within 72 h of admission from 104 patients admitted to hospital with COVID‐19. The results of the haematological and coagulation tests are in agreement with previous studies, showing that prolonged PT and APTT and raised D‐dimer, increased neutrophil counts and reduced lymphocyte counts are associated with mortality in these patients. Our data also show that raised sTM, and to a lesser extent D‐dimer, TAT and tPAI‐C at admission, are also associated with increased mortality. Moreover, sTM was independent of D‐dimer, TAT and PIC, and showed a greater discriminatory function than any of the other tests studied. In multiple logistic regression, increasing sTM was the only parameter which significantly associated with mortality.

Several other studies have shown increased plasma levels of sTM in COVID‐19 patients. In a study of 60 PCR confirmed COVID‐19 patients, sTM, von Willebrand factor and soluble endothelial protein C receptor were significantly higher in patients with severe disease.^13^ In the same study, immunohistochemistry in five post‐mortem samples, showed the loss of sTM expression in alveolar capillaries, compared to non‐COVID‐19 pulmonary disease controls. A prospective study of 63 COVID‐19 patients found significantly higher sTM levels in ICU than non‐ICU COVID‐19 patients. Those with moderate to severe acute respiratory distress had significantly higher sTM than patients with mild ARDS^6^ A study of samples collected from 49 COVID‐19 patients within 72 h of hospitalization, showed increased VWF and sTM in COVID‐19 patients relative to controls.^14^ In a longitudinal study of 14 COVID‐19 patients, high mortality was characterized by inhibition of fibrinolysis and higher sTM levels.^9^ Finally, a study of “long COVID” in 50 convalescent COVID‐19 patients, reported that endothelial biomarkers including VWF and sTM were significantly higher than normal controls, with a sustained endotheliopathy being more frequent in older, comorbid patients and those requiring hospitalization.^15^

The present study has several advantages over previous publications: we used fully automated CLEIA measurement, which can generate results within 20 min, whereas previous studies used ELISA techniques for sTM, which are time‐consuming and do not lend themselves to urgent testing of small sample numbers marker assays; a relatively large number of patients were studied which has provided more robust data; and all samples were taken within 72 h of admission.

The limitations of our study should be acknowledged. As the samples were anonymised, only limited clinical information was available on the patients. Due to the pressures of the pandemic on the coagulation laboratory, samples were not collected from consecutive patients.

Our data support growing evidence that endothelial dysfunction plays an important role in the pathophysiology of COVID‐19, and is associated with poor outcomes.^5^, ^16^, ^17^ While D‐dimer may identify patients who will benefit from therapeutic‐dose LMWH,^18^ others have found D‐dimer to be non‐discriminatory.^19^ It is thought that targeted interventions such as RAS inhibitors or statins may improve outcomes in patients with endothelial damage, by reducing NFκB (and hence tissue factor) expression and attenuating the effects of vascular endothelial growth factor.^16^, ^20^ Of the haemostatic variables measured, sTM, which can be rapidly assayed, is the best independent predictor of mortality in patients hospitalized with COVID‐19, and this suggests that endothelial dysfunction plays an important role in disease progression. We believe that rapid measurement of haemostatic activation markers, particularly sTM, may be useful in guiding personalized medicine for patients with COVID‐19.

## CONFLICT OF INTERESTS

CG is a consultant for Sysmex UK. IJM is a consultant for Sysmex Corp. PM and SP have no conflicts of interest to declare.

## AUTHOR CONTRIBUTIONS

CG, IJM, SP and PM designed the study. CG and SP prepared and analysed the samples. CH wrote the first draft of the manuscript; all authors contributed to the review and revision of the manuscript.

## Data Availability

The data that support the findings of this study are available from the corresponding author upon reasonable request.
